# Suspected Mitochondrial Dysfunction and Complex Pathophysiology in Fatal Hypermobile Ehlers–Danlos Syndrome: Insights from a Case Report and Post-Mortem Findings

**DOI:** 10.3390/biomedicines13020469

**Published:** 2025-02-14

**Authors:** Arash Shirvani, Purusha Shirvani, Ugochukwu Jonah, Brian E. Moore, Michael F. Holick

**Affiliations:** 1 Ehlers-Danlos Syndrome Clinical Research Program, Section of Endocrinology, Diabetes, Nutrition and Weight Management, Department of Medicine, Boston University Chobanian & Avedisian School of Medicine, Boston, MA 02118, USA; hn@bu.edu (A.S.); shirvani@bu.edu (P.S.); 2 The Department of Pathology & Laboratory Medicine at Boston Medical Center and Boston University Chobanian & Avedisian School of Medicine, Boston, MA 02118, USA; ugochukwu.jonah@bmc.org (U.J.); brian.moore@bmc.org (B.E.M.)

**Keywords:** Hypermobile Ehlers–Danlos Syndrome (hEDS), mitochondrial dysfunction, gastrointestinal dysmotility, Alzheimer’s Type II astrocytes, ATP6, m.9055G>A, impaction, postmortem, connective tissue disorder, multi-system complications

## Abstract

**Background/Objectives:** Hypermobile Ehlers–Danlos Syndrome (hEDS) is a complex connective tissue disorder with multi-systemic manifestations that significantly impact quality of life. This case report investigates the clinical course and molecular mechanisms of advanced hEDS through an in-depth case study and post-mortem findings. **Methods:** The clinical history of a 24-year-old patient with advanced hEDS was analyzed, focusing on progressive complications across multiple systems. Post-mortem examination and genetic analysis were performed to elucidate the underlying pathophysiology. **Results:** The patient’s clinical course was marked by gastrointestinal, neurological, and immune complications requiring numerous surgical interventions. Post-mortem findings revealed severe gastrointestinal dysmotility and Alzheimer’s Type II astrocytes. Genetic analysis identified variants in mtDNA genes ATP6, CYB, and ND, suggesting a potential role of impaired mitochondrial function in hEDS pathogenesis but requiring further validation through functional studies. **Conclusions:** This case report provides valuable insights into the potential role of mitochondrial dysfunction in advanced hEDS and highlights the need for further research in this area. Future studies should include comprehensive functional assays, longitudinal tissue sampling, family genetic analyses, and muscle biopsies to better understand the complex interplay between genetic factors, mitochondrial function, and clinical manifestations in hEDS. Establishing genetic bases and developing targeted therapies addressing both structural and metabolic aspects are crucial. The patient’s legacy offers invaluable information that could significantly contribute to enhancing diagnostic accuracy and developing personalized treatment strategies for this challenging disorder, potentially leading to better care for individuals living with hEDS.

## 1. Introduction

Hypermobile Ehlers–Danlos Syndrome (hEDS) is a complex inherited connective tissue disorder characterized by joint hypermobility, skin hyperextensibility, and tissue fragility [1]. EDS has been estimated to affect approximately 1 in 5000 individuals globally [2]. A study conducted in Wales found a combined occurrence of hEDS and Hypermobility Spectrum Disorder (HSD) of 1 in 500 individuals, with a notable gender disparity as 70% of those diagnosed were female [3]. hEDS follows an autosomal dominant inheritance pattern [4]. However, the severity and manifestations can vary significantly even within families [1,4]. Despite its relatively high prevalence compared to other EDS subtypes, hEDS remains underdiagnosed due to its clinical variability and lack of awareness among healthcare providers. Unlike other subtypes of EDS, such as the vascular or classical forms, hEDS lacks a clearly defined genetic mutation, making diagnosis and management particularly challenging [1,2,3,4,5]. This diagnostic uncertainty has led to cases of misdiagnosis, including false allegations of child abuse or Munchausen Syndrome by Proxy (MSbP), which delay appropriate care and cause significant emotional harm to families [6,7]. While hEDS primarily affects the musculoskeletal system, it also manifests as multi-systemic involvement, including gastrointestinal, immunological, cardiovascular, and neurological complications [1,5,8,9,10]. These complications significantly increase morbidity and require a multidisciplinary approach to care [5].

Despite advancements in understanding some aspects of hEDS, the molecular mechanisms driving its progression and multi-system dysfunction remain poorly understood. This knowledge gap, combined with the lack of definitive diagnostic tests, has contributed to cases where children with hEDS have been wrongly removed from their families due to misdiagnosis of their symptoms such as abuse or MSbP [6,7]. Emerging research points to abnormalities in extracellular matrix (ECM) components, particularly collagen, as key contributors to the pathophysiology of hEDS [11]. Dysregulation in ECM remodeling enzymes such as matrix metalloproteinases (MMPs) has also been implicated in tissue fragility and dysfunction across multiple organ systems [12]. Additionally, recent studies have suggested that metabolic disturbances, dysautonomia, and immune dysregulation may exacerbate disease progression, especially in advanced cases [13].

The aim of this case report is to present an in-depth case study of Karen Richards, a remarkable 24-year-old patient with advanced hEDS, whose clinical trajectory was characterized by severe multi-system complications and who made the selfless decision to donate her body to research to advance the understanding of hEDS.

**Figure 1:**
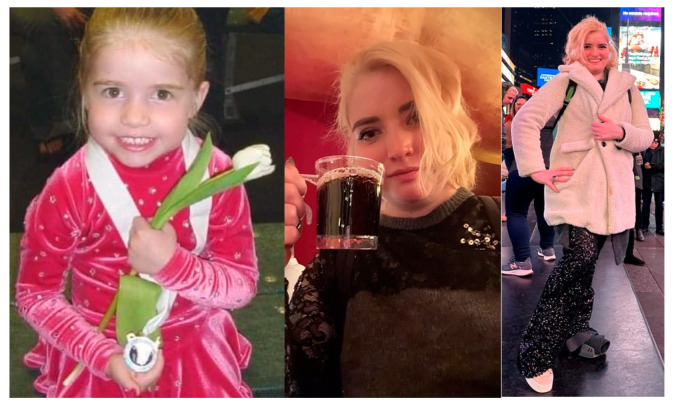
Karen Richards’ journey with Complex Hypermobile Ehlers–Danlos Syndrome (hEDS): A legacy of hope. Photographs documenting Karen’s life journey from childhood through early adulthood, showcasing the progression of her complex hEDS manifestations. Her dedication to advancing the medical understanding of hEDS through the selfless donation of her body to research exemplifies her commitment to helping future generations affected by this condition. This photograph is included with permission from Karen’s mother to honor their altruism, humanize the face of hEDS, and raise awareness about this challenging condition. By sharing her story visually, we hope to foster empathy among clinicians and improve the understanding of hEDS.

Karen’s case emphasizes the importance of thorough medical evaluations and underscores the need for improved awareness and diagnostic accuracy in distinguishing organic conditions like hEDS from fabricated or induced illnesses [6,7,14]. Her generous gift to science represents hope for developing better diagnostic tools and treatments for future generations affected by hEDS (Figure 1).

## 2. Case Report: Clinical Course of Advanced Hypermobile Ehlers–Danlos Syndrome (hEDS)

This section provides an overview of the clinical course of a patient with advanced hEDS while detailing key diagnoses, surgical interventions, challenges in symptom management, and how various complications interacted with one another over time.

### 2.1. Patient Background

The patient was a 24-year-old female diagnosed with advanced hEDS, a subtype of Ehlers–Danlos Syndrome characterized by joint hypermobility, skin hyperextensibility, and tissue fragility. Her medical history began at age 7 with a partial thyroidectomy for a large benign tumor at MD Anderson Cancer Center, marking the beginning of a complex medical journey spanning nearly two decades. Her medical history was marked by significant complications across multiple organ systems, including the gastrointestinal (GI), neurological, and cardiovascular systems. The interplay between gastrointestinal dysmotility, neurological issues including dysautonomia, EDS migratory pain syndrome, and recurrent infections created a cycle of complications that compounded over time. She experienced a series of debilitating conditions that severely impacted on her quality of life. Managing the patient’s complex array of symptoms posed significant challenges for her healthcare team.

### 2.2. Primary Diagnoses, Treatments, and Surgical Interventions

#### 2.2.1. Hypermobile Ehlers–Danlos Syndrome

The suspicion of EDS was first raised at age 9 due to recurrent shoulder instability, ligament laxity, and joint hypermobility. However, a formal diagnosis was not established at that time. By age 10, clinical notes documented joint laxity and hyperextensibility, and while EDS was considered, it was ultimately deemed an unlikely diagnosis. During this period, the patient also presented with chronic pain syndrome and was diagnosed with psychological factors affecting physical condition, leading to treatment for conversion disorder. At age 11, EDS became a more prominent consideration in her clinical evaluations after rheumatological disorders and mixed connective tissue disorders were excluded. By age 14, her hypermobility was formally documented using the Beighton Score, where she achieved a maximum score of 9/9, further supporting the diagnosis of EDS. The patient’s diagnosis of hEDS was officially confirmed at age 17 following the publication of the 2017 International Classification for EDS [1]. This timeline illustrates the complexities and challenges in diagnosing hEDS, particularly in young patients with multiorgan involvement.

hEDS served as the underlying condition that necessitated multiple surgical interventions throughout the patient’s life. At age 11, an initial ankle pinning for dislocation proved unsuccessful, leading to a more extensive ankle reconstruction with cadaver ligaments at age 12. The following year required a right shoulder reconstruction with allografts. The surgical notes documented right scapula prominence with posterior fullness, and the humeral head was palpable posterior to the glenoid. Post-procedure imaging confirmed proper shoulder reduction, and she was fitted with a custom shoulder sling featuring a soft plastic component designed to provide additional scapular stabilization.

The surgical team documented their concerns regarding compliance with immobilization guidelines, given her ability to readily redislocate the joint in what appeared to be a voluntary manner. This complication necessitated modified treatment approaches and highlighted the challenges in managing joint instability in hEDS patients.

#### 2.2.2. Progressive Spinal and Vascular Complications

**Figure 2:**
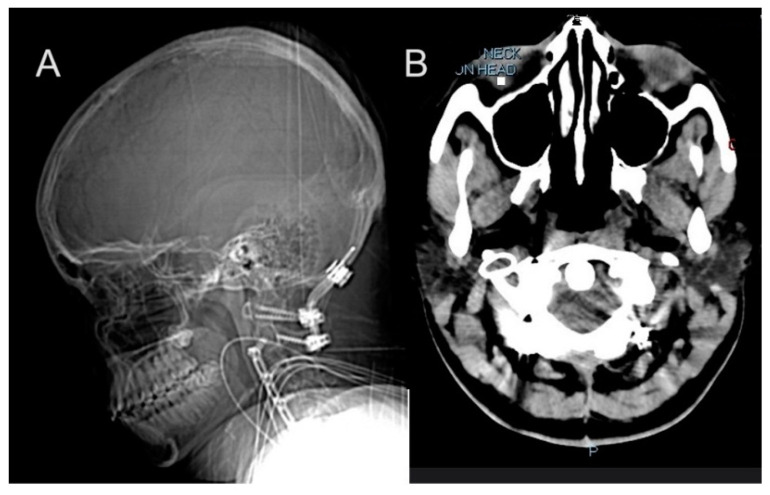

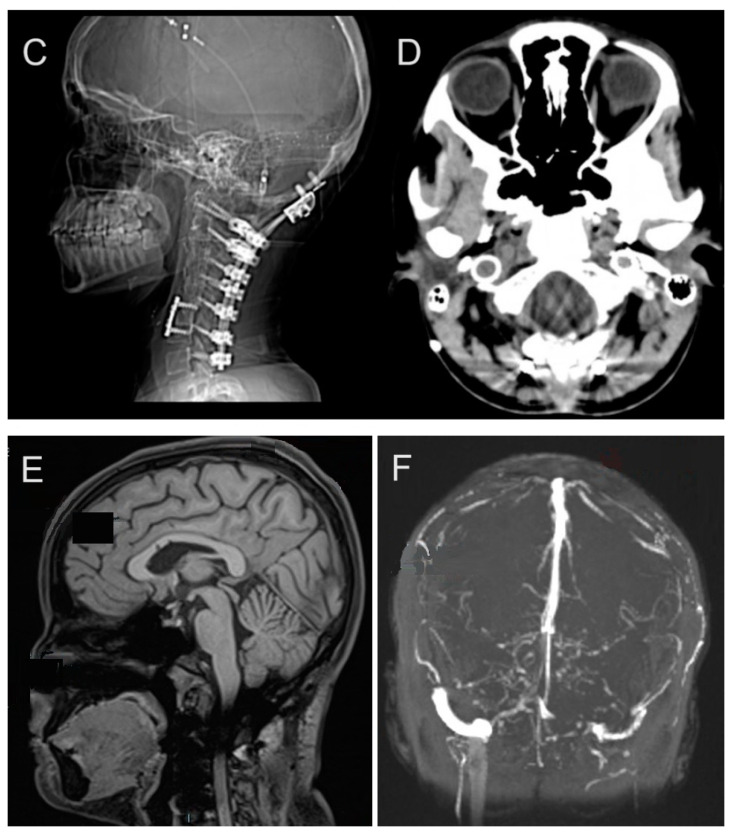
Longitudinal imaging findings in a patient with hEDS demonstrate progressive cervical spine instability and vascular complications. (**A**,**B**) A 2015 CT scan at age 15 showing post-surgical changes including occipital-C2 fusion and C2–C5 ACDF, with cervical lordosis straightening and C5–C6 anterolisthesis. Prior interventions included tethered cord release at age 12 and lumbar shunt placement at age 14 for IIH management. (**C**,**D**) CT in 2019 revealing expanded occipital-to-C7 fusion after hardware removal, necessitated by progressive cervical instability. (**E**) Brain MRI in 2016 demonstrates normal intracranial findings without restricted diffusion, hemorrhage, or tonsillar ectopia. (**F**) A 2016 angiogram showing asymmetric jugular veins with right-sided dominance, post-jugular foramen stenosis, and diminished left-sided flow.

At age 12, she underwent tethered cord release surgery, which was complicated by multiple cerebrospinal fluid (CSF) leaks requiring blood patches and surgical revisions. This was followed by a series of spinal surgeries beginning with a skull-C2 fusion in 2013. Her vascular surgical history included multiple stenting procedures beginning in response to jugular vein stenosis, a condition linked to impaired blood flow from the brain with right internal jugular and transverse sinus stent placement. Despite these interventions, she continued to experience symptoms related to venous insufficiency and intracranial pressure. Idiopathic Intracranial Hypertension (IIH) management began at age 14 with initial ICP monitoring and lumbar shunt placement. This condition resulted in chronic headaches and required multiple surgical interventions. In 2014, she received a lumboperitoneal (LP) shunt placement, which was later removed in 2015 due to a crack in the tubing and followed by C2-C5 anterior cervical discectomy and fusion (ACDF) in 2015 (Figure 2A,B). This period also marked her initial diagnosis of pseudotumor cerebri. The following year, in 2016, she required left internal jugular stent for thrombus and obstruction, followed by superior sagittal sinus stent placement in 2017. These interventions were necessitated by recurring thrombosis and vascular complications associated with her hEDS. By 2019, further deterioration required additional surgeries including O-C7 fusion with removal of anterior hardware in June (Figure 2C,D) and C6/7 ACDF in December. Three separate repairs were required to address CSF leaks from the lumbar shunt removal site. The management continued to evolve, with additional procedures including sagittal sinus stent placement at age 17 and multiple VP shunt revisions.

The spinal interventions progressed in complexity, culminating in a repair from occipital to T5 at age 20. These procedures were necessitated by progressive cervical instability and neurological symptoms. By 2022, imaging revealed significant hardware-related complications, including screw lucency at C7 and adjacent pedicle fracture. She developed myofascial neck pain secondary to multiple fusion procedures, ultimately requiring surgical wound washout and debridement. In April 2022, she underwent ultrasound-guided aspiration of a posterior cervical fluid collection, removing 90cc of brownish/purulent fluid, followed by surgical wound washout and debridement.

Each surgery presented unique challenges due to tissue fragility in hEDS patients, and many required subsequent revisions or additional procedures due to complications or failure of initial interventions.

#### 2.2.3. Psychological and Psychosocial

The patients’ psychological challenges began early in her medical journey, reflecting the profound emotional impact of managing advanced hEDS. By age 7, following her thyroidectomy and initial diagnosis of complex medical conditions, she began to experience anxiety related to her chronic illness and its unpredictable nature. This anxiety intensified as her symptoms progressed, leading to heightened stress by her early teens when she required multiple surgical interventions for joint instability and GI issues. At age 10, she developed pseudoseizures—a neuropsychological manifestation often induced by stress—further complicating her medical and emotional profile. Coping challenges became apparent around age 11, as her reliance on adaptive devices and medical support increased, reflecting the emotional strain of adjusting to her physical limitations.

Social and emotional isolation emerged as a significant issue by age 14, coinciding with her need for educational accommodations and frequent hospitalizations that limited her interaction with peers. Additionally, she began experiencing sleep disturbances in her mid-teens, around age 15, with the diagnosis of obstructive sleep apnea and fragmented sleep patterns. These issues likely contributed to fatigue-related mood disturbances, including irritability and reduced emotional resilience, further impacting her social and emotional well-being. Neuropsychological assessments during this period revealed autonomic nervous system dysregulation, dissociative episodes, and potential trauma responses, underscoring the profound psychological impact of her chronic condition.

Her case underscores the importance of addressing emotional health through multidisciplinary approaches, ensuring that psychological well-being is integrated into the broader care framework for patients with complex chronic conditions.

#### 2.2.4. Dysphagia and Vocal Cord Paralysis

The condition emerged as a critical complication at age 20, primarily resulting from structural issues related to improperly sized surgical hardware. Treatment approaches evolved over time, beginning with conservative management through speech therapy and swallow studies. As the condition progressed, more invasive interventions became necessary. The structural nature of the problem was particularly challenging because the problematic hardware could not be removed due to EDS-related complications.

#### 2.2.5. Gastroparesis and Dysautonomia

The patient experienced gastroparesis and dysautonomia from an early age, significantly impacting her gastrointestinal (GI) motility and quality of life. Gastroparesis, characterized by delayed gastric emptying without mechanical obstruction, was first suspected in childhood due to persistent symptoms of nausea, vomiting, bloating, and severe constipation. Dysautonomia, involving dysfunction of the autonomic nervous system, further exacerbated these symptoms and contributed to progressive GI dysmotility.

At the age of 9, the patient underwent abdominal radiography to evaluate obstruction and constipation. The imaging revealed a moderate to large amount of stool in the non-descended colon, confirming significant constipation. Two months later, an upper GI series with barium swallow was performed to investigate gastroesophageal reflux; the results were normal. By age 12, a gastric emptying study using a radiolabeled solid meal provided definitive evidence of gastroparesis, with a calculated gastric emptying T-1/2 of 189 min, indicating markedly delayed gastric emptying.

Initial management strategies focused on conservative measures, including dietary modifications and medications to improve gastric motility. Metoclopramide, a prokinetic agent, was utilized to stimulate smooth muscle contractions in the upper GI tract and facilitate gastric emptying. Additionally, probiotics were introduced to support gut health by modulating the intestinal microbiome, which may have been disrupted due to chronic dysmotility. Following probiotics, Magnesium Gluconate was administered as an osmotic laxative.

Several other medications were employed to manage specific symptoms associated with gastroparesis and dysautonomia while indirectly helping her constipation. Famotidine and Ranitidine HCl were used to address acid reflux symptoms often associated with GI dysmotility. Additionally, Levothyroxine Sodium was used to treat hypothyroidism-associated constipation by correcting thyroid hormone deficiency.

The patient also experienced symptoms consistent with irritable bowel syndrome with constipation (IBS-C) as well as Mast Cell Activation Syndrome (MCAS). To address this, Cromolyn Sodium, a mast cell stabilizer, was prescribed. This medication has been studied for its potential benefits in IBS-C by reducing symptoms such as bloating and abdominal discomfort associated with GI dysmotility.

As her condition progressed, more invasive interventions became necessary. By her late teens, she required enteral feeding through gastric and jejunal tubes due to severe nutritional deficiencies caused by her dysmotility. Despite these extensive efforts, the patient’s gastroparesis and dysmotility continued to worsen over time. By age 22, the patient required a series of feeding interventions, beginning with gastric tube placement, progressing to jejunal tube placement. By age 23, she sought alternative treatments in Sri Lanka, where she underwent Ayurvedic therapies aimed at improving GI motility alongside Western medical interventions. In her final year, attempts at Total Parenteral Nutrition (TPN) were unsuccessful due to complications such as sepsis. Each of these interventions presented its own complications, including tube malfunctions, infections, and absorption issues.

#### 2.2.6. Postural Orthostatic Tachycardia Syndrome (POTS)

Her POTS was diagnosed at the age of 16 in May 2016. Symptoms included tachycardia upon standing, dizziness, fatigue, and lightheadedness, which were exacerbated by underlying conditions such as hEDS. Management involved the use of Midodrine to address orthostatic hypotension and lifestyle modifications, including increased fluid and salt intake. However, complications arose due to overlapping medical issues including MCAS and neurogenic bladder, which added complexity to symptom control and required a multidisciplinary approach to care.

#### 2.2.7. Immune System Dysfunction and Recurrent Infections

Immune system dysfunction played a significant role in the patient’s medical history, particularly in the context of her diagnoses of MCAS and immunoglobulin deficiency, both of which are commonly associated with hEDS. These immune-related conditions were identified during her late teens and early twenties. Symptoms included recurrent allergic reactions, chronic hives, angioedema, idiopathic anaphylaxis, and increased susceptibility to infections, likely exacerbated by her connective tissue disorder. Management strategies involved antihistamines, mast cell stabilizers like cromolyn sodium, and epinephrine for severe allergic reactions. Additionally, she required immunoglobulin replacement therapy to address antibody deficiencies. Complications from these immune disorders included heightened inflammation, recurrent infections such as urinary tract infections and sepsis, and systemic effects that may have contributed to her other conditions, including POTS.

The patient experienced multiple episodes of bacteremia and sepsis, likely due to a combination of factors including compromised hEDS, immune function, chronic stress on multiple body systems, and possible IgG-3 deficiency. The frequency and severity of infections increased over time, particularly in her final months, contributing significantly to her overall decline. Urinary Tract Infections (UTIs) were first documented at age 17, with six infections occurring between December 2018 and January 2019 during a period of cervical spine compression. Management involved symptomatic treatments and neurosurgical evaluations for underlying causes. Sepsis episodes further complicated her condition. At age 22, she developed sepsis secondary to a polymicrobial infection in a posterior cervical fluid collection following spinal surgeries.

#### 2.2.8. Endocrine System

The patient had a complex endocrine history, including adrenal corticotropin hormone (ACTH) deficiency and hypothyroidism, both of which required ongoing management. ACTH deficiency (5 pg/mL; reference range: 9–57 pg/mL), diagnosed in September 2016 at the age of 16, resulted in secondary adrenal insufficiency, leading to symptoms such as fatigue, low blood pressure, and weakness. Management involved daily hydrocortisone therapy to replace cortisol levels and prevent adrenal crises, with adjustments during periods of physical stress or illness. The management of her endocrine dysfunction was further complicated by reactive hypoglycemia, which, while initially diagnosed alongside ACTH deficiency, showed improvement with cortisol supplementation.

Additionally, she underwent a partial thyroidectomy at age 7 due to a large benign tumor. Although clinically and biochemically euthyroid initially, she developed primary hypothyroidism by age 14, likely due to the progressive decline in function of the remaining thyroid tissue and increased hormonal demands during adolescence. This required subsequent levothyroxine therapy.

#### 2.2.9. Post-Operative and Compounding Complications

This case highlights the difficulties in managing advanced hEDS due to its broad symptomatology and multi-systemic impact. The interplay between GI dysmotility, neurological issues like IIH, and recurrent infections illustrates how complications can compound each other over time, leading to progressive deterioration despite extensive medical care. For example, the patient’s recurrent infections often led to increased intracranial pressure due to inflammation, which, in turn, exacerbated her IIH, and her neurological issues such as IIH were further complicated by the patient’s vascular problems, which impaired blood flow from the brain and contributed to increased intracranial pressure. GI dysmotility contributed to malnutrition and weakened immune function, making her more susceptible to infections.

Also, her extensive surgical history demonstrates the progressive nature of advanced hEDS and highlights the challenges in achieving long-term surgical success in patients with connective tissue disorders and, as a result, poor wound healing. Each intervention carried additional risks due to her underlying condition, often resulting in complications requiring further surgical management.

#### 2.2.10. Terminal Course

The terminal course of the patient’s condition, spanning ages 23 to 24, demonstrated the relentless progression of advanced hEDS and its multisystem complications. At age 23, she entered hospice care following a series of debilitating health challenges, including recurrent aspiration pneumonia, bacteremia, and severe GI dysmotility that rendered her unable to tolerate oral or enteral nutrition. Despite this, she sought alternative treatments in Sri Lanka, where she underwent Ayurvedic therapies aimed at improving GI motility alongside Western medical interventions. During this time, she experienced multiple complications, including shunt failure requiring replacement, two episodes of sepsis, cerebral deep vein thromboses (DVTs), peritonitis, and GJ tube failure necessitating replacement. While temporary improvements were observed during her time in Sri Lanka, these gains were short-lived as her condition deteriorated further.

Upon her return at age 24, the patient faced escalating health crises. Her dysphagia worsened to the point of intractable aspiration with all oral intake, confirmed by swallow studies showing aspiration across all liquid consistencies and a significant pharyngeal residue. These issues led to recurrent aspiration pneumonia and bacteremia, with three documented episodes in her final year. Attempts at TPN were unsuccessful due to complications such as sepsis. Consultations with leading centers like Mayo Clinic could not resolve her feeding intolerance or aspiration risks. Ultimately, she chose to re-enter hospice care and discontinue aggressive medical interventions, expressing exhaustion from living in a hospital setting.

### 2.3. Post-Mortem Findings

**Figure 3:**
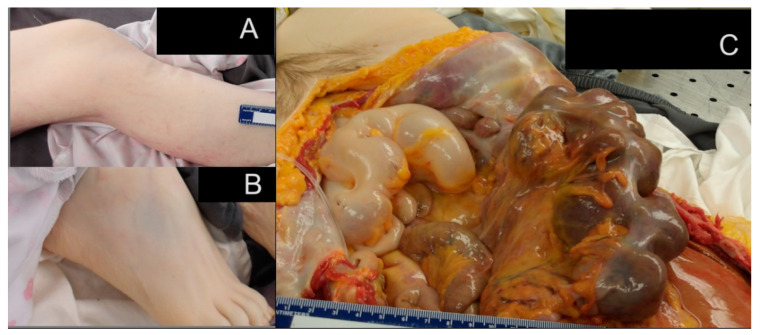
Post-mortem findings in a 24-year-old female with advanced hypermobile Ehlers–Danlos syndrome (hEDS). (**A**) Multiple stretch marks (striae) on the skin, especially over the chest, abdomen, and limbs. (**B**) Thin, translucent skin with visible underlying veins. (**C**) Distended bowel with fecal impaction and a gastrostomy tube in situ, suggestive of severe gastrointestinal dysmotility.

The post-mortem examination of this 24-year-old patient with advanced hEDS revealed several significant findings that provide valuable insights into the pathophysiology and progression of the disease. These findings span multiple organ systems and help elucidate the complex interplay between various complications in advanced hEDS. The body was that of an adult female measuring 162.5 cm in length (approximately 5 feet 4 inches) from the top of the head to the bottom of the heels. External features included a central venous line and a gastrostomy tube, with multiple stretch marks noted on the lower extremities; blue-tinged sclera; and thin, translucent skin with visible veins underneath and stretch marks (Figure 3).

#### 2.3.1. Gastrointestinal System Findings

The most striking post-mortem finding was severe GI dysmotility, evidenced by severe constipation with inspissated stool occupying approximately 70% of the large bowel. This finding is particularly significant as it demonstrates the severe impact of connective tissue dysfunction on GI motility in advanced hEDS. The affected bowel showed areas of thinned-out, pale wall tissue, suggesting chronic distention and possible compromised blood flow. The presence of both gastrostomy and enterostomy tubes at autopsy further highlighted the severity of the nutritional challenges faced during life.

#### 2.3.2. Central Nervous System Findings

**Figure 4:**
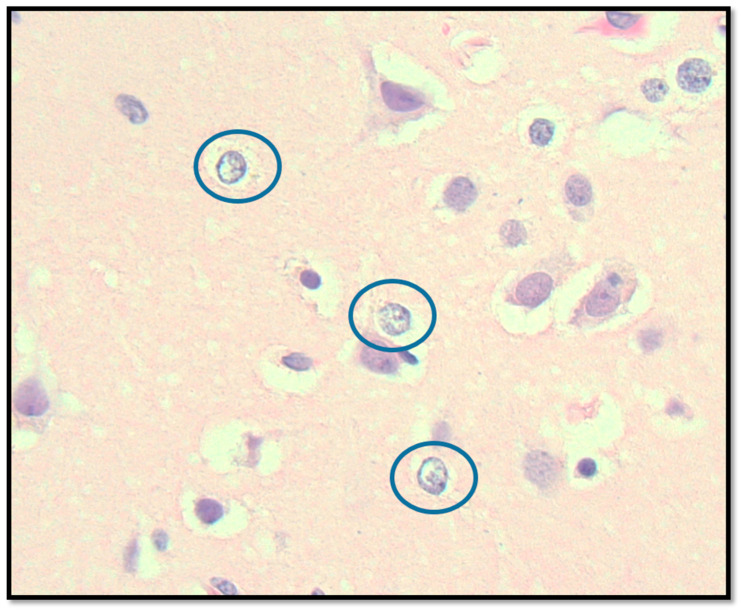
Cerebral neocortical histopathology demonstrating scattered Alzheimer Type II astrocytes. Photomicrograph (400× magnification) showing astrocytes with characteristic nuclear chromatin clearing (circles) in the cerebral neocortex, a finding associated with metabolic encephalopathy, without other significant pathological changes.

The brain weight was 1300 g, within normal limits, with no evidence of herniation or focal lesions. However, microscopic examination revealed the presence of Alzheimer Type II astrocytes (Figure 4), a significant finding suggesting hepatic encephalopathy. This observation is particularly interesting as it indicates metabolic disturbances that may not be commonly recognized in hEDS patients.

#### 2.3.3. Cardiovascular System Findings

Despite the patient’s extensive history of vascular complications, including multiple DVTs and various stenting procedures, the post-mortem examination of the cardiovascular system was surprisingly unremarkable. The heart weighed 300 g, within normal limits, and showed no evidence of coronary artery disease or myocardial infarction. This is particularly noteworthy given her history of multiple jugular vein stenting procedures, recurrent DVTs, and other vascular complications requiring intervention. The absence of significant cardiovascular pathology at autopsy suggests that the vascular complications experienced during life may have been primarily functional rather than structural. This could be related to factors such as autonomic dysfunction, inflammatory processes, altered vascular reactivity, or complex interactions between connective tissue abnormalities and vascular function.

#### 2.3.4. Additional Significant Findings

Additionally, a microscopic focus of fibrosis with lymphocytic infiltrate was found in the left lung, which may represent prior infection or a secondary inflammatory response but is not characteristic of hEDS.

#### 2.3.5. Multifactorial Causes of Death

The patient passed away at 4:00 a.m. on 11 August 2024 due to a complex cascade of medical complications stemming from her hEDS. The immediate cause of death was determined to be bowel obstruction, which may lead to metabolic derangements and ultimately systemic failure. The autopsy was performed 11 h postmortem at Boston Medical Center. 

## 3. Molecular Mechanisms Underlying Multi-System Dysfunction in Advanced hEDS

The fundamental pathophysiology of hEDS involves complex alterations in connective tissue structure and function. While the specific genetic mutations underlying hEDS remain elusive, unlike other EDS subtypes, the disorder clearly affects the synthesis, processing, and organization of collagen and other extracellular matrix (ECM) components [4,15]. These abnormalities lead to widespread tissue fragility and dysfunction across multiple organ systems. In hEDS fibroblasts, gene expression changes related to cell–matrix interactions and inflammatory responses contribute to the complex pathogenesis [15]. The ECM disorganization observed in hEDS cells may be a consequence of excessive pathological turnover, mainly due to ECM-degrading enzymes and other unknown factors [15]. Specifically, fibroblasts show disorganization of collagens and fibronectin, with notable changes in integrin receptor expression patterns [15,16]. The αvβ3 integrin is preferentially recruited due to the lack of fibronectin-ECM and its canonical integrin receptor [15]. Recent research suggests that the defective ECM has consequences for cellular processes, with hEDS fibroblasts exhibiting impairments in cell adhesion and cytoskeleton organization [16]. Pathogenesis involves alterations in membrane-bound collagen and aberrations in cell adhesion and cytoskeleton dynamics that could drive the abnormal properties of the connective tissue [16]. These molecular changes include altered integrin expression profiles that affect cell adhesion, cell signaling, and cell survival through the formation of focal adhesions and signaling complexes [16]. In hEDS fibroblasts, the αvβ3 integrin transduces signals to the ILK-Snail1-axis, inducing a fibroblast-to-myofibroblast transition [15,17,18]. The irregular collagen network and resulting tissue softness leads to altered mechanotransduction due to reduced ECM stiffness, disrupted cellular organization, and increased sensitivity to mechanical stimuli [19]. These molecular changes create a feedback loop where ECM disorganization leads to further cellular dysfunction and inflammatory responses, contributing to the complex pathogenesis of hEDS [15,16,17,18,19].

### 3.1. Neurological Complications and Blood–Brain Barrier (BBB) Dysfunction

The pathophysiology of neurological complications in hypermobile hEDS involves complex interactions between extracellular matrix disruption and BBB dysfunction [20]. Collagen abnormalities associated with connective tissue disorders can lead to neuroinflammation and weaken the integrity of the BBB [21,22,23]. The compromised ECM architecture affects both BBB and meningeal integrity through multiple interconnected pathways, leading to neuroinflammatory responses and increased risks of neurological disorders [1,3,20].

Mast cells play a central role in this process, with nearly 97% located on the brain side of blood vessels [1,22]. When aberrantly activated, these cells release inflammatory mediators including histamine, proteases, and cytokines that increase BBB permeability and promote tissue remodeling [21,24]. The significant association between hEDS and MCAS—with approximately one-third of MCAS patients having comorbid hEDS—suggests a mechanistic link between these conditions [4,25]. Recent research has highlighted the role of β1-integrin-mediated adhesion of brain endothelial cells to the surrounding ECM in stabilizing claudin-5 in BBB tight junctions and maintaining BBB integrity [26]. The disruption of this interaction in hEDS may contribute to BBB dysfunction and subsequent neurological complications.

This creates a self-perpetuating cycle where ECM disruption leads to increased tissue fragility and permeability [27]. The compromised meningeal integrity, combined with altered vascular permeability and tissue mechanics, makes hEDS patients particularly susceptible to disorders of CSF pressure regulation [20]. Common manifestations include spontaneous CSF leaks and IIH [20].

The neurological manifestations can significantly impact daily functioning. Studies have shown that 67% of hEDS patients report headaches, with this number increasing to 95% in patients with concurrent MCAS [28]. Additionally, peripheral neuropathy is extremely common, with 97% of hEDS patients reporting chronic neuropathic pain [28].

Understanding these molecular mechanisms and their clinical implications is essential for developing targeted therapeutic approaches for managing neurological complications in hEDS patients. This includes addressing both the underlying connective tissue dysfunction and the associated inflammatory responses.

### 3.2. Mast Cell Activation Syndrome in hEDS

The relationship between hEDS and MCAS represents a complex interaction between connective tissue dysfunction and immune dysregulation [25]. Our recent genetic studies have identified variations in several genes including MT-CYB, HTT, MUC3A, HLA-B, and HLA-DRB1, which are implicated in both hEDS and associated mast cell activation disorders [25]. These genetic alterations affect pathways involved in antigen processing, mucosal protection, and collagen synthesis [25].

Mast cells, which are predominantly located in connective tissues and near blood vessels, contain granules with hundreds of different mediators including histamine, heparin, proteases, prostaglandins, leukotrienes, and various cytokines and chemokines [16,25,29]. In hEDS patients, the irregular collagen network and resulting tissue softness leads to mechanical instability that promotes excessive mast cell degranulation [19]. When aberrantly activated, these cells release inflammatory mediators that contribute to multiple system manifestations.

#### Gastrointestinal and Respiratory Complications

The GI system is particularly affected, with studies showing a significantly higher prevalence of symptoms in hEDS patients compared to controls: abdominal pain (69% vs. 27%), postprandial fullness (34% vs. 16%), and other digestive issues [30]. This may be explained by the high concentration of mast cells in the GI tract, where their activation leads to altered motility, increased permeability, and visceral hypersensitivity [30].

Respiratory manifestations, particularly dyspnea, dysphonia, asthma, sleep apnea, and reduced respiratory muscle function show a strong association with hEDS/MCAS [31]. Studies have demonstrated that modifications of matrix proteins in lung parenchyma, combined with mast cell-mediated inflammation, can alter biomechanics and repair responses [28,31]. This creates a cycle where tissue damage and inflammation perpetuate each other, leading to increased bronchial reactivity and airway remodeling [28,31].

### 3.3. Metabolic Dysfunction and Chronic Fatigue

The relationship between hEDS and metabolic dysfunction represents a complex interaction that may explain the high prevalence of fatigue in affected individuals. Recent research has revealed several key molecular mechanisms underlying this connection through longitudinal cytokine profiling and multi-omics analysis [32].

The molecular basis for fatigue in hEDS appears to involve several interconnected pathways. The irregular collagen network leads to increased mechanical stress and energy demands as muscles work to compensate for joint instability [33]. Additionally, the pro-inflammatory state observed in hEDS contributes to altered cellular metabolism and increased energy expenditure [33].

Recent research has identified potential roles for the BCL6 and TP53 pathways in disease etiology, suggesting involvement of fundamental cellular processes in energy metabolism and stress responses [32]. These pathways may explain the high prevalence of fatigue in hEDS patients, as the disruption of normal cellular energy production and stress responses could lead to systemic metabolic dysfunction [32]. Fibroblasts from hEDS patients demonstrate significant alterations in cellular metabolism and energy production [34]. These cells show changes in gene expression related to cellular metabolism, redox balance, and protein folding in the endoplasmic reticulum [34]. The altered metabolic state leads to increased energy expenditure and decreased efficiency in ATP production. The molecular basis for fatigue in hEDS appears to involve several interconnected pathways [34]. The irregular collagen network leads to increased muscle strain, as muscles work overtime to compensate for joint instability [35]. This constant compensation requires additional energy expenditure and leads to faster muscle fatigue [36]. Additionally, the pro-inflammatory myofibroblast-like phenotype observed in hEDS cells contributes to altered cellular metabolism and increased energy demands [15,34].

The metabolic dysfunction is further complicated by GI manifestations common in hEDS. Studies have shown significantly higher prevalence of GI symptoms that affect nutrient absorption and metabolism, with 86% of hEDS patients reporting GI symptoms including dysphagia (14.3%), gastroesophageal reflux (52.4%), and malabsorption issues [37]. This creates a cycle where poor nutrient absorption leads to decreased energy availability, contributing to chronic fatigue. Recent proteome analysis has revealed changes in proteins involved in cellular metabolism and redox balance, suggesting that mitochondrial dysfunction may play a role in the fatigue experienced by hEDS patients [34]. Our recent research has also identified specific genetic variants affecting mitochondrial function through the OXPHOS system, including genes such as MT-CYB, MT-ND1, EMC1, and ACAD9 [25]. This suggests a potential role for mitochondrial dysfunction in the pathogenesis of both hEDS and MCAS, contributing to the complex manifestations of these conditions [25]. These metabolic alterations, combined with chronic pain and poor sleep quality, create a complex pattern of energy depletion that contributes to the persistent fatigue characteristic of hEDS [38].

### 3.4. Genetic Variations and Biochemical Findings in the Patient

Comprehensive Metabolic Panel (CMP) and Amino Acid Testing were performed at Methodist Children’s Hospital of South Texas, San Antonio, TX. Blood serum analysis revealed low potassium (3.2 mmol/L; reference range: 3.5–5.2 mmol/L) and low ALT (19 U/L; reference range: 25–65 U/L), potentially indicating electrolyte imbalance and nutritional or metabolic factors, respectively. Anion gap (16.2; reference 10–20) and chloride levels (107 mmol/L; reference 98–109 mmol/L) were normal. Copper levels in urine were normal, ruling out Wilson’s disease as a cause of metabolic derangements. Urine amino acid testing demonstrated decreased levels of several branched-chain amino acids: valine (39 μmol/g Cr; reference range: 70–171 μmol/g Cr), methionine (34 μmol/g Cr; reference range: 38–147 μmol/g Cr), and isoleucine (21 μmol/g Cr; reference range: 26–105 μmol/g Cr). These findings are more likely attributable to impaired nutrient absorption and/or altered renal handling of amino acids rather than a direct indicator of mitochondrial dysfunction. A subsequent metabolic panel showed normal results except for elevated glucose (139 mg/dL; reference range: 70–99 mg/dL), and the amino acid profile revealed elevated alanine (769 nmol/mL; reference range: 152–547 nmol/mL). While elevated plasma alanine can be associated with mitochondrial disorders due to increased glycolysis, it may also be influenced by other factors such as metabolic stress.

MECP2 Mutation Analysis and Chromosomal Microarray were conducted at Children’s Hospital Boston, DNA Diagnostic Laboratory, using blood genomic DNA. The results confirmed a normal female karyotype (46,XX) with no evidence of chromosomal abnormalities, low-level mosaicism, point mutations, deletions, or duplications in the tested regions. Additionally, genetic testing revealed no mutations in the MECP2 gene, ruling out Rett syndrome and related conditions.

Mitochondrial genome sequencing was performed using mtSEEK^®^ at Courtagen Diagnostics Laboratory, a CLIA-certified facility specializing in genetic and mitochondrial testing. This comprehensive test was designed to sequence the entire mitochondrial genome and detect deletions and heteroplasmy levels. The process involved extracting genomic DNA from a saliva sample, which was then amplified using specific primer sets for the mitochondrial genome. High-coverage sequencing (over 5000×) was performed using Illumina MiSeq technology, and heteroplasmy levels were quantified using a reference variant control set. The laboratory employed proprietary bioinformatics pipelines (Ziphyr^®^) to analyze sequencing data and measure heteroplasmy levels. The analysis identified the ATP6 m.9055G>A variant in homoplasmy, and the patient’s mitochondrial haplogroup was identified as K2a8, as detailed in Table 1.

### 3.5. Suspicion of Mitochondrial Involvement in This Patient with Advanced hEDS

**Figure 5:**
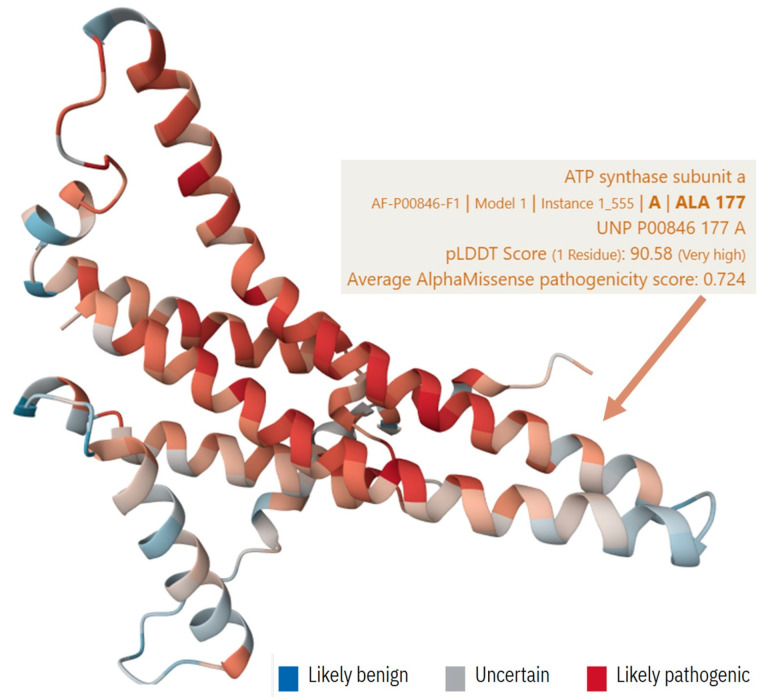
AlphaFold predicted structure of human ATP6 protein (P00846) with likely pathogenic Ala177Thr variation. This figure presents a predicted structural model of the ATP6 protein, generated using the AlphaFold tool [39]. The ATP6 protein is a critical component of the mitochondrial ATP synthase complex, essential for energy production in cells through oxidative phosphorylation. Notably, the patient in this study carried a specific variation in the ATP6 gene, where alanine (Ala) at position 177 is converted to threonine (Thr). This mutation is predicted to be likely pathogenic based on the average AlphaMissense pathogenicity score for all possible amino acid substitutions at this position. The pLDDT score for this region is very high, indicating high confidence in the structural prediction. The displayed color for each residue represents the average AlphaMissense pathogenicity score across all possible substitutions, providing insights into the potential impact of this mutation on protein function. This structural model helps elucidate how such variation could affect the protein’s overall architecture and its role in mitochondrial energy metabolism.

In this patient, the complex clinical presentation and limited genetic findings raise suspicion of potential mitochondrial dysfunction as a contributing factor to her advanced hEDS. While her genetic analysis did not reveal extensive mitochondrial DNA variations, the presence of specific variants, particularly the ATP6 m.9055G>A, suggests a possible role of mitochondrial dysfunction in hEDS (Figure 5).

This mitochondrial dysfunction hypothesis is supported by the observed blood lactic acidosis (3.7 mmol/L; reference range: 0.4–2.0 mmol/L) and elevated alanine, which is often associated with compromised cellular energy production [40]. Despite the exclusion of secondary causes of lactic acidosis through clinical assessments and laboratory findings, the evidence for mitochondrial dysfunction in this case remains circumstantial. It is important to note that while these findings suggest a potential role for mitochondrial dysfunction in this patient’s condition, they are insufficient to definitively diagnose a mitochondrial disorder. To strengthen this hypothesis, additional studies would be necessary, including muscle biopsy with histological analysis, respiratory chain complex activity measurements, and heteroplasmy analysis across different tissues. The secondary causes of lactic acidosis were systematically evaluated and excluded through clinical assessments and laboratory findings. These included hypoxia-related conditions, inborn errors of metabolism, endocrine dysfunctions, renal or hepatic failure, and drug-induced effects.

## 4. Discussion

Despite increasing recognition of hEDS, effective targeted therapies remain scarce, and the long-term consequences of this condition are often poorly understood. This case report, detailing the clinical journey and post-mortem findings of a patient with advanced hEDS, provides critical insights into the systemic manifestations, potential genetic underpinnings, and the complex interplay of factors contributing to disease progression. 

The autopsy findings revealed severe intestinal dysmotility, which is consistent with the GI complications frequently reported in hEDS [41]. Patients with hEDS often experience a range of GI symptoms, including abdominal pain, bloating, nausea, constipation, diarrhea, and gastroparesis, which significantly impact their quality of life [41]. The pathophysiology likely involves multiple factors, including structural abnormalities in smooth muscle tissue due to defective collagen/matrix, autonomic dysfunction affecting gut motility, chronic inflammation, and tissue remodeling, and possible involvement of the enteric nervous system [42,43]. These findings underscore the importance of aggressive management of GI symptoms in hEDS patients, as severe dysmotility can contribute significantly to morbidity and mortality [44]. The extensive clinical documentation spanning nearly two decades, combined with detailed post-mortem findings and comparative analyses, offers valuable perspectives on the intricate interplay between connective tissue dysfunction and mitochondrial pathology. The patient exhibited genetic variations in several mitochondrial genes, including ATP6, ND1, ND2, ND5, CO3, and CYB. While most variants were classified as benign or likely benign, the ATP6 mutation was of uncertain significance or likely pathogenic (Table 1). A recent study suggests that specific combinations of common mtDNA variants, previously considered benign, may contribute to mitochondrial impairment [45]. This dysfunction can influence ECM gene expression [45], potentially explaining some connective tissue abnormalities observed in hEDS. Furthermore, histamine-activated mitochondrial ROS production has been shown to exacerbate mitochondrial dysfunction [29,45], which may be particularly relevant in patients with MCAS often associated with hEDS [29]. Notably, these findings point to a novel genetic mechanism for mitochondrial disorders, where the interaction of common, non-pathogenic mtDNA variants leads to functional incompatibility and subsequent mitochondrial dysfunction [45].

The patient’s lactic acidosis, combined with her clinical manifestations, potentially supports mitochondrial dysfunction. Despite ruling out secondary causes of lactic acidosis, the evidence for mitochondrial dysfunction remains indirect. The complex interplay between the patient’s mitochondrial DNA variations and her multisystem clinical presentation underscores the need for further investigation into potential mitochondrial involvement.

While the observed biochemical abnormalities and genetic findings suggest a possible role for mitochondrial dysfunction in this patient’s condition, they are insufficient for a definitive diagnosis of a mitochondrial disorder. To strengthen this hypothesis, future studies should include muscle biopsy with histological analysis, respiratory chain complex activity measurements, functional assays to evaluate mitochondrial performance, and heteroplasmy analysis across different tissues, particularly muscles. These additional investigations would provide more conclusive evidence to elucidate the extent of mitochondrial dysfunction and its role in the pathophysiology of hEDS.

The presence of the ATP6 mutation typically suggests Leigh or NARP (Neuropathy, Ataxia, and Retinitis Pigmentosa) syndrome, which occurs in over 50% of patients with MT-ATP6 mutations [46,47,48]. While the patient’s lactic acidosis supports this diagnosis, other aspects of the presentation are atypical [44,49]. A recent genomic study of 566 EDS patients revealed 79 mitochondrial DNA changes in genes including MT-ND, CYB, CO, and ATP6, suggesting mitochondrial dysfunction may contribute to EDS. In this case, the genetic profile, combined with clinical and biochemical presentation, suggests a possible overlap syndrome where mitochondrial dysfunction may contribute to or modify the expression of hEDS [50]. Overlap in MT-ATP6 pathogenic variant heteroplasmy level is observed between some asymptomatic individuals and others with NARP or mild neurologic manifestations, particularly for variants other than m.8993T>G [49,50,51,52].

The presence of Alzheimer’s Type II astrocytes in the brain parenchyma of this patient with advanced hEDS is a fascinating finding that warrants careful consideration. While these modified astrocytes are classically associated with hyperammonemia secondary to liver dysfunction, the patient’s medical history and post-mortem liver histology revealed no overt evidence of hepatic encephalopathy or significant liver pathology. Therefore, the etiology of these astrocytic changes in this case remains unclear. While subtle metabolic derangements not detectable by routine liver function tests or histological examination, altered cerebral amino acid metabolism specific to hEDS, or increased permeability of BBB cannot be ruled out, the presence of Alzheimer’s Type II astrocytes in this hEDS patient may also be related to underlying mitochondrial dysfunction. Further research is needed to elucidate the potential significance of this observation in hEDS, including investigating the role of mitochondrial function and other potential contributing factors.

The hypothetical mitochondrial dysfunction may explain the heightened mast cell reactivity observed in our patient, as adequate ATP levels are essential for proper mast cell function and FcεRI-dependent activation [53]. Mitochondria are actively involved in the FcεRI-dependent activation of mast cells [53]. The connection between mitochondrial dysfunction and immune dysregulation is further supported by the observation that patients with MT-ATP6 variants demonstrate increased susceptibility to infections and immune-mediated complications, similar to the high prevalence of MCAS and recurrent infections in the reported hEDS patient [54]. Notably, a recent study found evidence of tissue-specific effects of mitochondrial variants [47], which may explain the variable manifestation of symptoms across different organ systems in hEDS patients. The impaired oxidative phosphorylation and energy production resulting from these mitochondrial variants could contribute to the chronic inflammation and tissue fragility characteristics of hEDS, creating a vicious cycle where energy deficiency leads to compromised tissue repair and heightened immune responses. These findings suggest that potential therapeutic approaches targeting mitochondrial function might be beneficial in managing both the structural and immunological aspects of hEDS. Studies have demonstrated that phosphodiesterase-5 (PDE5) inhibitors may benefit cases with increased mitochondrial membrane potential, while α-ketoglutarate and aspartate supplementation could help support cellular energy metabolism [55,56]. Future clinical trials may consider tailoring therapeutic approaches to address both mitochondrial dysfunction and tissue-specific manifestations.

Recent studies have revealed promising therapeutic approaches targeting mitochondrial dysfunction in neurodegenerative disorders [55,56]. PDE5 inhibitors have shown promise in cases with increased mitochondrial membrane potential, as demonstrated by significant improvements in neural progenitor cells [47,55]. Additionally, metabolic support through α-ketoglutarate and aspartate supplementation may help optimize cellular energy production by providing key substrates for the citric acid cycle and supporting mitochondrial function [56]. Successful management requires coordination between multiple healthcare providers, including primary care physicians, physical therapists, occupational therapists, pain specialists, mental health professionals, and relevant subspecialists [10]. Patient education is fundamental and should cover understanding the condition and its manifestations, joint protection strategies, activity modification techniques, warning signs requiring medical attention, and self-management strategies [57]. The evidence supports the need for a patient-centered approach that considers the various manifestations of the condition and their impact on daily life [58].

The diagnosis of hEDS remains primarily clinical, with no specific genetic test currently available, making it essential for clinicians to follow the 2017 International Classification criteria, which emphasizes the assessment of generalized joint hypermobility, systemic manifestations, and family history [1]. Early diagnosis enables timely intervention and prevents potential complications, though the path to diagnosis can be lengthy and complex [8].

This case report has several significant limitations that must be considered when interpreting its findings. As a single case report, the observations and conclusions drawn may not be generalizable to the broader hEDS population. The patient’s unique genetic profile and complex medical history may represent an extreme presentation of the condition. A major limitation is the lack of direct functional evidence to confirm mitochondrial dysfunction. While we identified mtDNA variants and observed biochemical alterations that could be consistent with mitochondrial impairment, these findings alone are insufficient to establish the diagnosis of a complex mitochondrial disorder. We did not perform crucial functional studies such as muscle biopsy with detailed histological analysis, RCC activity measurements, or other direct assessments of mitochondrial function. The absence of these key functional assays limits our ability to conclusively establish the role of mitochondrial dysfunction in the patient’s condition.

The retrospective nature of the analysis limits our ability to establish causal relationships between observed phenomena. The lack of longitudinal tissue samples throughout the patient’s life course restricts our understanding of disease progression at a cellular level. Furthermore, the absence of a comprehensive family history and genetic analysis of relatives limits our ability to fully elucidate the hereditary aspects of the patient’s condition.

Despite these limitations, this case report provides valuable insights into the potential role of mitochondrial dysfunction in advanced hEDS and highlights the need for further research in this area. Future studies should include comprehensive functional assays, longitudinal tissue sampling, family genetic analyses, and muscle biopsies to better understand the complex interplay between genetic factors, mitochondrial function, and clinical manifestations in hEDS.

The establishment of comprehensive molecular diagnostic criteria and genetic panels for hEDS would serve multiple crucial purposes: providing definitive diagnosis, preventing the mischaracterization of symptoms as MSbP [6,8], protecting families from false allegations of abuse [59,60,61,62], and enabling timely and appropriate medical interventions that could be life-saving. This underscores the urgent need for continued research into the genetic basis of hEDS, the development of reliable diagnostic tools, and the implementation of personalized therapeutic approaches that address both the structural and metabolic aspects of the condition.

## 5. Conclusions

The case of Karen Richards, a 24-year-old patient with advanced hEDS, provides unprecedented insights into the complex pathophysiology of this challenging disorder. Through her selfless donation to research, a comprehensive analysis of her clinical course, genetic profile, and post-mortem findings revealed a critical new understanding of the molecular mechanisms underlying hEDS. The identification of mitochondrial variants, particularly the ATP6 mutation, alongside multiple mitochondrial DNA variations, suggests a previously unrecognized role of cellular energetics in disease progression.

Most importantly, Karen’s case serves as a powerful reminder of the human cost of misdiagnosis and delayed intervention in complex genetic disorders like hEDS. The absence of definitive molecular diagnostic tools continues to place patients at risk for inappropriate diagnosis of conditions like Munchausen Syndrome by Proxy, potentially leading to devastating consequences for families. Her legacy, through the scientific insights gained from her case, provides hope for developing more accurate diagnostic approaches and targeted therapeutic strategies. The interplay between connective tissue dysfunction, mitochondrial impairment, and immune dysregulation observed in her case underscores the necessity for a multidisciplinary approach to patient care and highlights the urgent need for improved diagnostic criteria and clinical awareness of hEDS. Karen’s contribution to medical science will undoubtedly influence the future direction of research and clinical care, offering hope for improved outcomes for patients and families affected by this complex disorder.

## Figures and Tables

**Table 1:** Genetic variants associated with the patient’s mitochondrial haplogroup.

Nucleotide Change	Amino Acid Changes	Coding Context	Gene Name	ALFA Allele Frequency	Manifestation	dbSNP ID
m.3480A>G	p.Lys58Lys	c.174G>A	MT-ND1	G = 0.09	Homoplasmy	rs28358584
m.4561T>C	p.Val31Ala	c.92C>T	MT-ND2	C = 0.02	Homoplasmy	rs41376350
m.9055G>A	p.Ala177Thr	c.529A>G	MT-ATP6	A = 0.15	Homoplasmy	rs193303045
m.9698T>C	p.Leu164Leu	c.174G>A	MT-CO3	C = 0.15	Homoplasmy	rs9743
m.9716T>C	p.Gly170Gly	c.510C>T	MT-CO3	C = 0.018	Homoplasmy	rs41502750
m.10550A>G	p.Met27Met	c.81G>A	MT-ND4L	G = 0.09	Homoplasmy	rs28358280
m.11299T>C	p.Thr180Thr	c.540C>T	MT-ND4	C = 0.14	Homoplasmy	rs28358285
m.11467A>G	p.Leu236Leu	c.708G>A	MT-ND4	G = 0.25	Homoplasmy	rs2853493
m.11719G>A	p.Gly320Gly	c.960A>G	MT-ND4	A = 0.59	Homoplasmy	rs2853495
m.12308A>G	n/a *	n/a	MT-TRNL2	G = 0.21	Homoplasmy	rs2853498
m.12372G>A	p.Leu12Leu	c.36A>G	MT-ND5	A = 0.25	Homoplasmy	rs2853499
m.14167C>T	p.Glu169Glu	c.507A>G	MT-ND6	T = 0.15	Homoplasmy	rs193302977
m.14766C>T	p.Thr7Ile	c.20T>C	MT-CYB	T = 0.65	Homoplasmy	rs193302980
m.14798T>C	p.Phe18Leu	c.52C>T	CYTB	C = 0.16	Homoplasmy	rs28357681

* This variant is located in the MT-L2 gene, which encodes for mitochondrial tRNA-Leucine.

## Data Availability

The data presented in this study are available on request from the corresponding author. Interested researchers who wish to access the data should contact the corresponding author directly with their request. Data sharing will be considered on a case-by-case basis, subject to the approval of the patient’s family and in compliance with institutional guidelines and applicable regulations governing human-subject research.
